# Pan-drug, colistin, streptomycin, erythromycin, clindamycin resistant *Salmonella enterica* serovars isolated from slaughtered cattle and human in mansoura, Egypt

**DOI:** 10.1186/s12941-025-00809-4

**Published:** 2025-07-03

**Authors:** Shimaa El Baz, Hanan Ahmed Zaher, Wafaa Ragab

**Affiliations:** 1 Department of Hygiene and Zoonoses, Faculty of Veterinary Medicine, Mansoura University, Mansoura, 35516 Egypt; 2 Department of Food Hygiene, Safety and Technology, Faculty of Veterinary Medicine, Mansoura University, Mansoura, 35516 Egypt; 3 Department of Bacteriology, Immunology and Mycology, Faculty of Veterinary Medicine, Mansoura University, Mansoura, 35516 Egypt

**Keywords:** *Salmonella*, Multidrug-resistant, Virulence genes, Antibiotic resistance, Abattoir workers

## Abstract

**Objectives:**

*Salmonella* is recognized globally as a significant foodborne pathogen associated with foodborne outbreaks in both humans and animal. The rise of multidrug-resistant (MDR) *Salmonella* isolates poses a critical public health challenge. Given that the isolation of *Salmonella* within abattoirs is a prominent source of community infection especially through the consumption of contaminated meat. This study aims to determine the prevalence of *Salmonella*, the occurrence of virulence genes (*invA*, *spvC*), and specific resistance genes (*tetA*, *sul1*, *aadA1*, *aac(3)- IV*) in *Salmonella* isolates isolated from cattle in abattoirs. Additionally, the investigation assesses the potential exposure risks for abattoir workers in Mansoura City, Egypt.

**Methods:**

In a study conducted from May to July 2024, a total of 150 samples were collected to investigate the presence of *Salmonella* in healthy Egyptian Baladi cattle and abattoir workers at the Mansoura abattoir, Mansoura City, Egypt. The sample collection comprised rectal swabs (*n* = 50) and meat swabs (*n* = 50) from cattle, in addition to 50 hand swabs obtained from abattoir workers. *Salmonella* isolation was done following standard microbiological techniques. Initially, pre-enrichment of the samples was conducted using buffered peptone water. Subsequently, selective enrichment was executed using Rappaport Vassiliadis broth, followed by cultivation on xylose-lysine-deoxycholate (XLD) agar to isolate suspected *Salmonella* colonies. These colonies were then subjected to a series of identification tests, including biochemical assays, slide agglutination tests, and polymerase chain reaction (PCR) targeting the *invA* gene, which is indicative of *Salmonella* presence. Furthermore, molecularly identified isolates were tested for the virulence gene *spvC*, which is related to the pathogenicity of *Salmonella*. The antimicrobial susceptibility of the isolates was assessed using the Kirby-Bauer disc diffusion method, providing insight into the resistance profiles of the observed isolates. In addition, a subset of 19 *Salmonella* isolates underwent multiplex PCR analysis to evaluate the presence of specific resistance genes: *tetA*, *sul1*, *aadA1*, and *aac(3)-IV*.

**Results:**

The overall occurrence of *Salmonella* isolates across all examined samples was 12.7%. This included 4% from cattle carcass swabs, 12% from rectal swabs, and a notable 22% from workers’ hands. The most prevalent serotypes identified were *Salmonella* Enteritidis and *Salmonella* Typhimurium, exhibiting incidences of 26.3% (*n* = 5) and 21% (*n* = 4), respectively. Other serotypes included *Salmonella* Infantis at 15.8% (*n* = 3), *Salmonella* Kentucky and *Salmonella* Tsevie each at 10.5% (*n* = 2), and *Salmonella* Paratyphi A, *Salmonella* Haifa, and *Salmonella* Virchow at 5.3% ((*n* = 1) each). From the tested *Salmonella* isolates, 100% (19/19) were positive for the *invA* and 89.5% (17/19) carried *Spvc* genes. Resistance profiling categorized the isolates into pandrug-resistant (PDR) at 5.3%, extensively drug-resistant (XDR) at 5.3%, multidrug-resistant (MDR) at 63.1%, and low drug-resistant at 26.3%. Notably, *Salmonella* Enteritidis exhibited complete resistance to all tested antimicrobial agents, resulting in a Multiple Antibiotic Resistance (MAR) index of 1. Conversely, *S.* Typhimurium was classified as XDR, with a MAR index of 0.937. Resistance rates were alarmingly high, with 100% against streptomycin, 89.5% against erythromycin, 73.7% against clindamycin, and 63.2% against ampicillin. Among the resistance genes, the *aadA1* gene was most prevalent at 100%, followed by *sul1* and *tetA* at 42.1% (*n* = 8) and 31.6% (*n* = 6), respectively. The *aac(3)-IV* gene was the least prevalent, noticed in 15.8% (*n* = 3) of the isolates.

**Conclusion:**

The high occurrence of multidrug-resistant (MDR) *Salmonella* serovars among the tested isolates is concerning. These antibiotics are crucial for effectively treating severe salmonellosis, highlighting the crucial need for strict antimicrobial regulation in both veterinary and human medicine.

**Supplementary Information:**

The online version contains supplementary material available at 10.1186/s12941-025-00809-4.

## Introduction

Salmonellosis persists as the second most prevalent bacterial foodborne illness in the European Union. In 2023, EU surveillance systems identified 77,486 confirmed human cases and the majority of infections were attributed to serovars: *Salmonella* Enteritidis, *Salmonella* Typhimurium, *Salmonella* Infantis and *Salmonella* Coeln [1]. Nontyphoidal *Salmonella* species cause the highest rates of illness and mortality worldwide, particularly in Europe. They are mainly transmitted through infected food and water, with *Salmonella enterica* subspecies *enterica* being the most significant for humans and domestic animals [2, 3].

*Salmonella* is often found in cattle, particularly in dairy cows, which serve as reservoirs for *Salmonella enterica* [4]. Human cases of salmonellosis typically arise from consuming infected poultry, eggs, dairy products, and beef [5]. The occurrence of *Salmonella* in cattle can contaminate milk and meat on farms, resulting in both direct and indirect infections in humans and other animals [6]. Infected animals may show symptoms of illness or shed *Salmonella* in their feces without exhibiting any clinical signs. The challenge of asymptomatic carriers is that these cattle can introduce the bacteria into abattoirs, which poses a considerable food safety risk due to the potential for cross-contamination during food processing [7]. Salmonella can persist in the environment for several months and is commonly linked to wildlife, which acts as a reservoir and source of infection.

Currently, there are approximately 2,700 *Salmonella* serotypes [8]. In cows, various *Salmonella* species are present, with serovars such as *Salmonella* *Dublin*, *Salmonella Newport*, and *Salmonella Typhimurium* being the most common. Additionally, asymptomatic carriers of *Salmonella* serotypes, including *Salmonella Cerro*, S*almonella Kentucky*,* Salmonella Mbandaka*, and *Salmonella Montevideo*, are commonly found in dairy animals through fecal shedding [9]. Many virulence factors are incriminated in the pathogenicity of *Salmonella* species for instance, the invasion A (*invA*) gene, the enterotoxin gene (*stn)*, and the *Salmonella* plasmid virulence C protein gene (*spvC*) [10].The *invA* gene helps in the adhesion and invasion of *Salmonella* to host epithelial cells of the intestinal mucosa, while the *spvC* gene promotes survival within these cells, aids in systemic invasion, and reduces cytokine production. Both genes are essential for assessing the pathogenicity of *Salmonella* isolates [11].

The global emergence of antimicrobial-resistant NTS presents a critical public health threat [12]. Surveillance data reveal alarming multidrug resistance (MDR) rates, with studies reporting 53.8% MDR among NTS isolates in Vietnam and Taiwan (2014–2019) [13], 47% in China, and 50% in sub-Saharan Africa [14, 15]. Tetracyclines and sulfonamides are broad-spectrum antibacterial agents that have been used for over fifty years to treat bacterial infections. However, their widespread use has contributed to the development of resistance in various bacteria, with Salmonella exhibiting particularly high rates of resistance to both tetracycline and sulfonamide [16]. Studies show extremely high resistance to tetracycline in poultry-associated isolates (98–100%), clearly linking this problem to antibiotic use in farming [13]. Similarly, about 43% of clinical Salmonella isolates are resistant to sulfonamides [17]. These findings highlight how antibiotic overuse in both human medicine and agriculture contributes to the development of resistant bacteria.

Aminoglycosides are vital veterinary antimicrobials used to treat infections in all major food-producing animals, and the World Health Organization (WHO) classifies them as vital for human medicine as well. The massive use of antibiotics in animal food production has resulted in an increase in antimicrobial-resistant isolates of *Salmonella* [18]. Therefore, the current study aimed to assess the occurrence of *Salmonella* isolates identified from cattle carcass swabs, rectal swabs, and hand swabs of abattoir workers in Egypt. Additionally, it focused on the identification of virulence genes (specifically *invA* and *spvC*) and antimicrobial resistance profiles (including *tetA*,* sul1*,* aadA1*, and *aac(3)-IV*), highlighting the potential public health hazards related to the transmission of multidrug-resistant (MDR) *Salmonella*.

## Materials and methods

### Study area and period

**Fig. 1:**
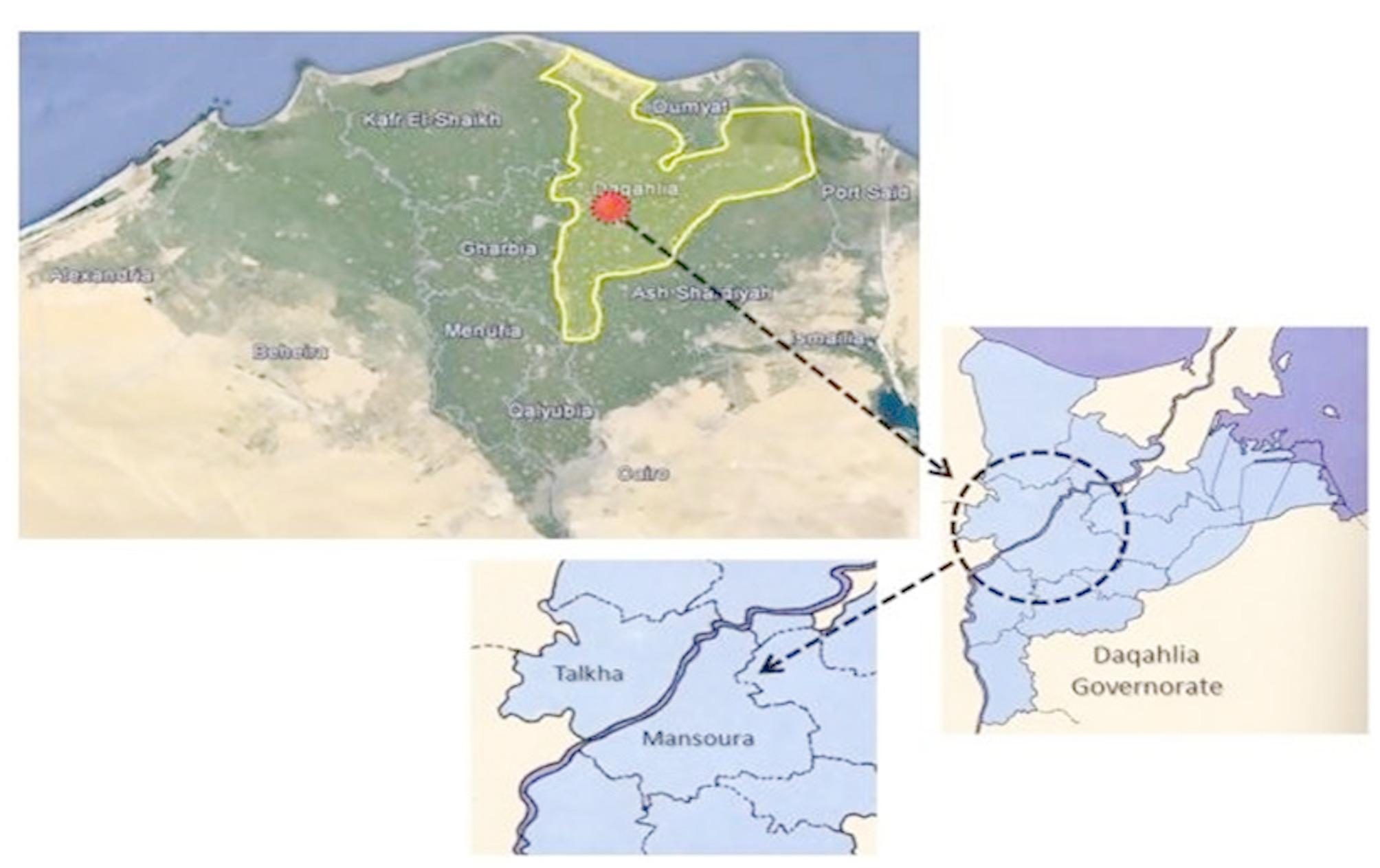
Map of the study area

This study was conducted from May to July 2024 at a large traditional slaughterhouse in Mansoura City, Egypt Figure (1). At the Mansoura abattoir, approximately 700 heads of cattle and sheep are slaughtered each week. The slaughtering process typically takes place after sunrise, between 6 a.m. and 11 a.m. Butchers carry out the slaughtering, while veterinarians perform ante-mortem and post-mortem inspections. Trained workers manage other related activities. The animals for slaughter are sourced from various nearby areas of Mansoura city and transported by truck, with differing treatment conditions until they reach the slaughter operation. The animals were randomly selected and divided into two groups: one group was slaughtered as usual, while the other group was allowed a period of rest and treated gently before slaughter. The animals were slaughtered by hand in a horizontal position on the floor before being lifted for 5 to 6 min to ensure complete bleeding. This was followed by skinning and evisceration in the same area within the slaughterhouse.

### Study design

A cross-sectional study was carried out to evaluate the prevalence and antimicrobial susceptibility of *Salmonella* in slaughter cattle and their contacts at the Mansoura abattoir.

### Sample collection, preparation, isolation and identification technique

We randomly selected fifty clinically healthy cattle (Egyptian Baladi breed, 3–4 years old) at slaughter. From each animal, we collected paired rectal (antemortem) and carcass swabs (*n* = 100 total: 50 rectal, 50 meat). Carcass samples were obtained from three anatomical sites (abdomen, round, and breast) using sterile cotton-tipped swab (2 × 3 cm) was moistened in buffered peptone water (BPW; CM0509B; Oxoid Ltd., Basingstoke, UK) and thoroughly rubbed over each designated area both horizontally and vertically several times, following the International Organization for Standardization [19]. In addition, 50 hand swabs (swabbing the palm of the right hand) were collected from the workers at the abattoir in Mansoura City, Egypt. All samples were placed on ice and transported to the laboratory for further analysis within 5 h.

To enrich cecal samples, approximately 1 gram of cecal contents was mixed with 10 ml of buffered peptone water (BPW; Oxoid, Basingstoke, UK). The enrichment mixture was then underwent incubation at 37 °C for 24 h. Following incubation, 0.1 ml of the cultured buffered peptone water was added to 10 ml of Rappaport-Vassiliadis broth (RV; Oxoid Ltd., Basingstoke, UK) and kept in an incubator at 42 °C for 20 to 24 h. Following this incubation, a single loop from each enriched broth was spread onto selective solid media, xylose lysine-desoxycholate (XLD) agar (Oxoid, Basingstoke, UK). The streaked plates were then incubated at 37 °C for another 24 h. All presumptive *Salmonella* colonies, recognized by their pink color with or without a black center on XLD agar, were selected and transferred to Tryptone Soya agar plates (Oxoid Ltd., Basingstoke, UK).

### Biochemical identification

The presumptive colonies were then tested biochemically using the API 20E system (BioMérieux, Marcy-l’Étoile, France) aided in identifying *Salmonella* based on the biochemical features of the isolates [20].

### Serological identification

All PCR-confirmed isolates (19) of *Salmonella* were typed using the Kauffman-White scheme [21] through a slide agglutination test to identify Somatic (O) and flagellar (H) antigens with polyvalent and monovalent *Salmonella* antiserum (DENKA SEIKEN Co., Japan). Briefly, a suspension of the organism’s growth was mixed in 0.5 ml of saline solution. On a microscopic slide, one drop of *Salmonella* Polyvalent “O” antiserum was placed in one circle, and a drop of saline (negative control) in another. We added one drop (0.05 ml) of the bacterial suspension to each circle, mixing gently for 1–2 min while avoiding evaporation. A positive reaction was indicated by rapid, complete agglutination, while delayed or partial agglutination was considered negative.

### Screening of virulence and resistance genes

#### Genomic DNA extraction

The identified bacterial colonies were cultivated on XLD agar (Oxoid, Basingstoke, UK) and then transferred to Luria-Bertani (LB) broth (Oxoid, UK), where they were incubated overnight at 37 °C. DNA was obtained from the bacterial cultures in the broth using the GeneJET Genomic DNA Purification Kit (Germantown, MD, USA) according to the manufacturer’s instructions.

#### Polymerase chain reaction (PCR) for identification of virulence and resistance genes

The amplification procedure was carried out using a Thermal Cycler (Master Cycler, Eppendorf, Hamburg, Germany). The sequence, product size and annealing temperature of all forward and reverse primers used are detailed in Table (1). All primers were obtained from (Promega-USA). For detecting virulence genes (*invA* and *spvC*), a multiplex PCR reaction mixture of 25 µL was prepared with 2 µL of DNA template, 0.5 µl of each primer mix (10 pmol/ µL each), 5 µL of 5× PCR buffer, 2.5 µL of 25 mM MgCl₂, 0.5 µL of 10 mM dNTPs, and 14.2 µL of deionized water, along with 0.3 µL (1.5 U) of Taq DNA polymerase. The PCR conditions included initial denaturation at 94 °C for 2 min, followed by 30 cycles of 94 °C for 45 s, annealing at 53 °C for 1 min, and extension at 72 °C for 1 min, with a final extension at 72 °C for 7 min. For amplifying of antibiotic resistance genes (*tetA* (tetracycline resistance), *sul1* (sulfonamide resistance), *aadA1*, and *aac(3)-IV* (aminoglycosides resistance), multiplex PCR was conducted according as previously reported [22], a 25 µL multiplex PCR mixture was prepared with 0.5 µl of each primer mix (10 pmol/ µL each), 2 µL of DNA template, 5 µL of PCR buffer, 2.5 µL of 25 mM MgCl₂, 0.5 µL of 10 mM dNTPs, and 14.2 µL of deionized water, along with 0.3 µL (1.5 U) of Taq DNA polymerase. The PCR cycling conditions were defined as follows: initial denaturation at 94 °C for 5 min, followed by 30 cycles of amplification consisting of 5 s at 94 °C, 10 s at 68 °C, and 20 s at 72 °C, with a final extension at 72 °C for 7 min. All PCR-amplified fragments were resolved by gel electrophoresis using a 1.5% (w/v) agarose gel (Puregene™, India). stained with ethidium bromide solution (0.5 µg/ml). The fragments were visualized under an ultraviolet transilluminator (acculab, Montreal, Quebec, Canada) and then photographed.

**Table 1:** Primer sequences, target product sizes, and optimal annealing temperatures used for PCR amplification in this study

Target gene	Oligonucleotide sequence (5′ → 3′)	Annealing temperature	Product size (bp)	References
*invA* (F)	**5**′ TATCGCCACGTTCGGCAA ′**3**	53 °C for 1 min	275	Nayak et al. (2004) [23]
*invA* (R)	**5**′ TCGCACCGTCAAAGGAACC ′**3**			
*spvC* (F)	**5**′ CGGAAATACCATCAAATA ′**3**		669	Swamy et al. (1996) [24]
*spvC* (R)	**5**′ CCCAAACCCATACTTACTCTG ′**3**			
*tetA* (F)	**5**′ GCTACATCCTGCTTGCCTTC ′**3**	68 °C for 10 s	201	Jaja et al. (2019) [25]
*tetA* (R)	**5**′ CATAGATCGCCGTGAAGAGG ′**3**			
*sul1* (F)	**5**′ TCACCGAGGACTCCTTCTTC ′**3**		316	Randall et al. (2004) [22]
*sul1* (R)	**5**′ AATATCGGGATAGAGCGCAG ′**3**			
*aadA1* (F)	**5**′ TAT CAG AGG TAG TTG GCG TCAT ′**3**		484	Randall et al. (2004) [22]
*aadA1* (R)	**5**′ GTT CCA TAG CGT TAA GGT TTC ATT ′**3**			
*aac(3)-IV* (F)	**5**′ TTGCGATGCTCTATGAGTGGCTA ′**3**		627	Stoll et al. (2006) [26]
*aac(3)-IV* (R)	5′ CTCGAATGCCTGGCGTGTTT ′3			

#### Antibiotic susceptibility testing for *Salmonella* isolates

Antibiogram of 19 molecularly identified *Salmonella* isolates was assessed against sixteen clinically used antibiotics commonly administered in Egyptian dairy cow farms, as explained in Table (2). This testing utilized the disk-diffusion method on Mueller–Hinton agar (Oxoid, Basingstoke, UK). In brief, Four or five colonies from the XLD agar plate of each isolate were mixed by 2 ml of sterile water to make suspension of final concentration of 0.5 CFU/ml which is equivalent to a 0.5 McFarland standard [27]. After that, the antibiotic discs were spread on the inoculated plate, which was then underwent incubation at 37 °C overnight. Susceptibility, intermediate susceptibility, and resistance of isolates were determined following the guidelines provided by the Clinical and Laboratory Standards Institute [28]. The Multiple Antibiotic Resistance (MAR) index was estimated for all *Salmonella* isolates as the ratio of the number of antibiotics to which an isolate was resistant to the total number of tested antibiotics [29]. A MAR index greater than 0.2 implies a high risk of contamination and the potential for extensive antibiotic use.

**Table:** 

Antimicrobial class	Antimicrobial agent	Sensitivity disc concentration (ug)
Aminoglycosides	Streptomycin (S)	10
	Gentamicin (G)	10
	Amikacin (AK)	30
Macrolides	Erythromycin (E)	15
	Cefoxitin (CE)	30
	Azithromycin (AZ)	15
Lincosamide	Clindamycin (CL)	10
B-lactams	Ampicillin (AM)	10
	Imipenem (IPM)	10
Cephalosporins	Cefazolin (CZ)	30
	Cefotaxime (CF)	30
Sulphonamides	Sulfamethoxazole/ Trimethoprim (SXT)	25
Tetracycline	Tetracycline (T)	30
Polymyxin	Colistin (CO)	25
Quinolones	Ciprofloxacin (CP)	5

## Results

A total of one hundred and fifty samples were collected from the abattoir to isolate *Salmonella* serovars, consisting of 50 samples each of cattle carcass swabs, rectal swabs, and workers’ hands. All suspected isolates underwent serological identification, virulence detection targeting two virulence genes, *invA* and *spvC*, and antimicrobial susceptibility testing using the Kirby–Bauer disc diffusion method for 16 antimicrobial agents of 9 different antibiotic classes. Additionally, four resistance genes targeting aminoglycosides, tetracycline and sulfonamide resistance, including *aadA1*, *aac(3)-IV*,* tetA* and *sul1*, respectively were tested using PCR.

All suspected isolates underwent serological identification, virulence detection targeting two virulence genes, *invA* and *spvC*, and antimicrobial susceptibility testing using the Kirby–Bauer disc diffusion method for 16 antimicrobial agents of nine different antibiotic classes. Additionally, four resistance genes targeting aminoglycosides, tetracycline and sulfonamide resistance, including *aadA1*, *aac(3)-IV*, *tetA* and *sul1*, respectively, were tested using PCR.

### Prevalence and serotyping of the recovered *Salmonella*isolates

**Fig. 2:**
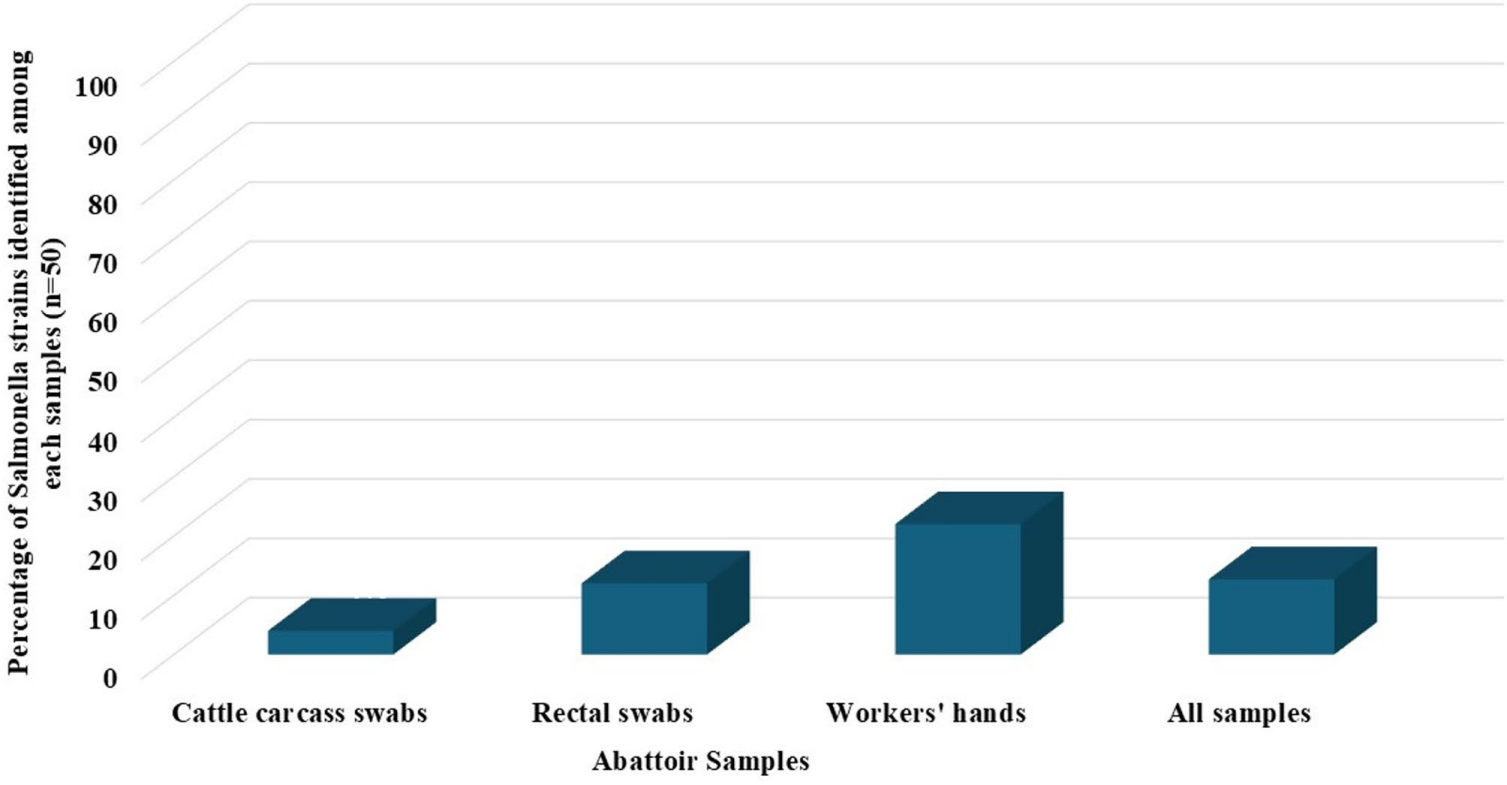
Distribution of *Salmonella* isolates (*n* = 19) recovered from cattle and human samples

**Fig. 3:**
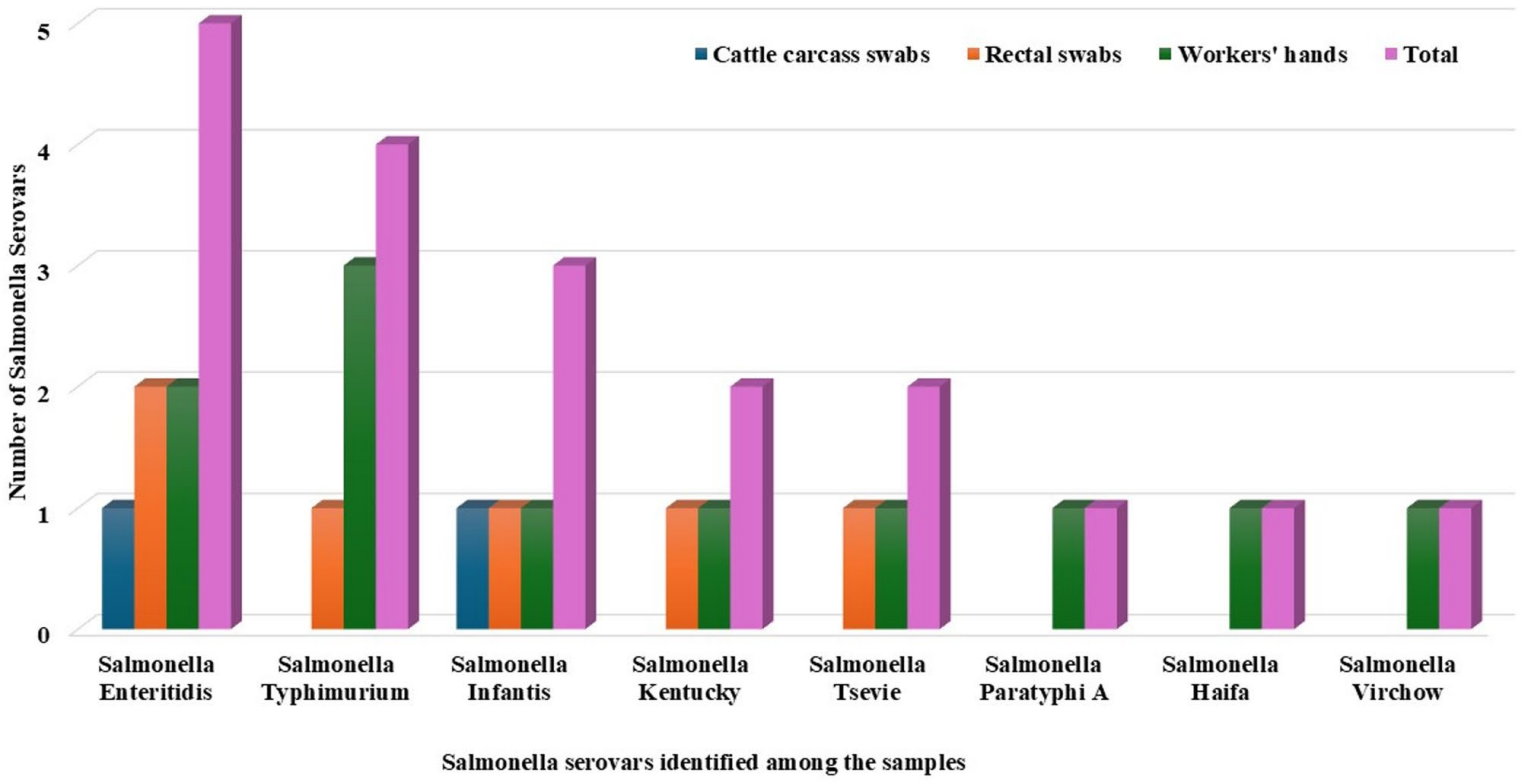
Serological identification of *Salmonella* isolates (*n* = 19) isolated from cattle and human samples

A total of 19 *Salmonella* isolates (12.7%) were obtained from 150 samples collected at the abattoir. These isolates included 4% (2/150) from cattle carcass swabs, 12% (6/150) from rectal swabs, and 22% (11/150) from workers’ hands (Fig. 2). The 19 *Salmonella* isolates were serologically identified into 8 different serotypes. *S.* Enteritidis and were the most prevalent serotypes, with incidences of 26.3% (5/19) and 21% (4/19), respectively. They were followed by *S.* Infantis at 15.8% (3/19), *S.* Kentucky at 10.5% (2/19), *S.* Tsevie at 10.5% (2/19), and *S.* Paratyphi A, *S.* Haifa, and *S.* Virchow, each at 5.3% (1/19) (Fig. 3).

### Antimicrobial susceptibility of *Salmonella* isolates

**Fig. 4:**
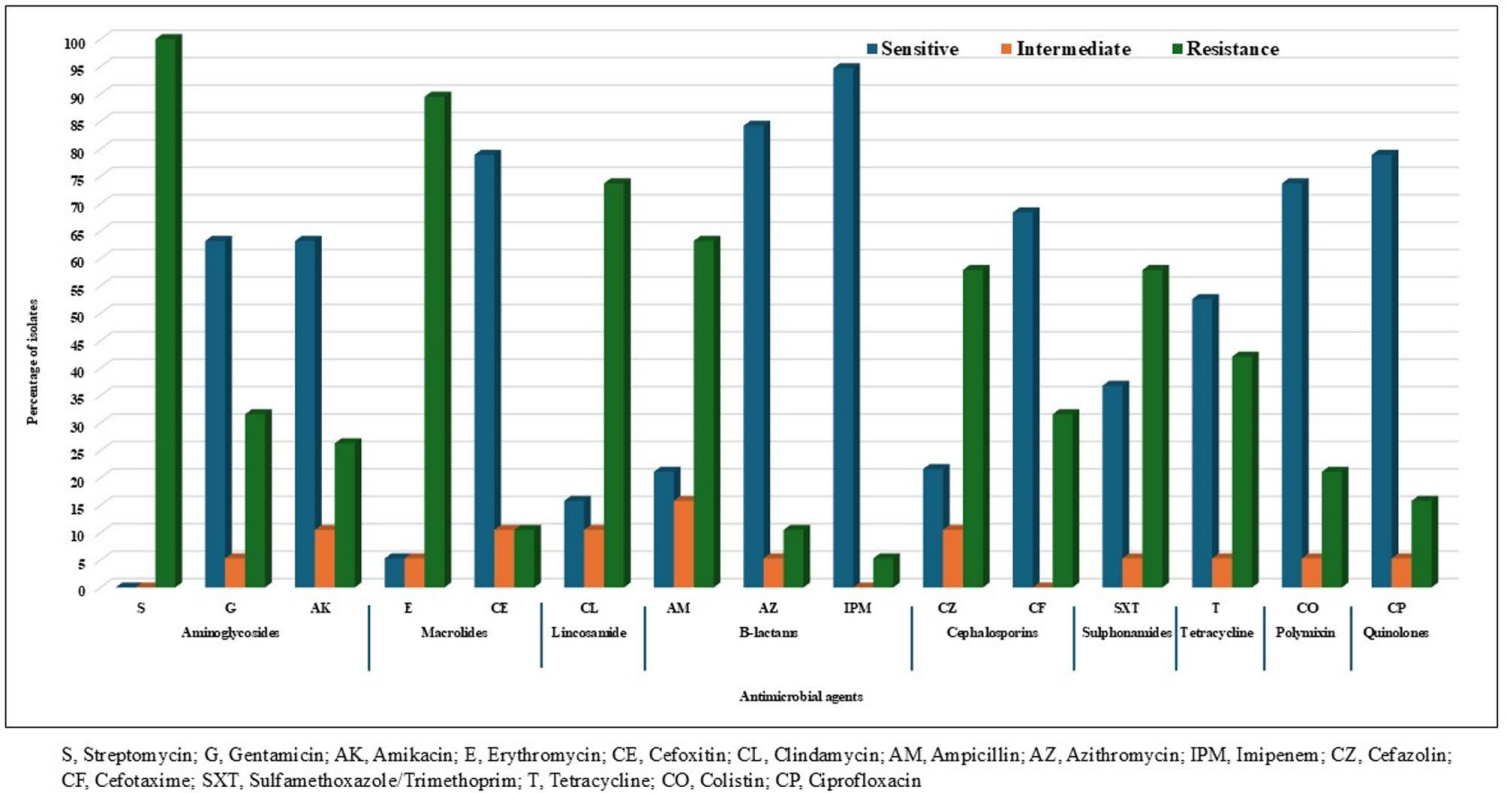
Antibiogram profiles of *Salmonella* isolates (*n* = 19) against 16 different antimicrobial agents

The 19 isolates of *Salmonella* isolates demonstrated a high resistance rate of 100% (19/19) against streptomycin, followed by 89.5% (17/19) against erythromycin, 73.7% (14/19) against clindamycin, and 63.2% (12/19) against ampicillin. Additionally, they exhibited moderate resistance rates of 57.9% (11/19) against both cefazolin and sulfamethoxazole, 47.4% (9/19) against neomycin, and 42.1% (8/19) against tetracycline. In contrast, the *Salmonella* isolates showed lower resistance rates of 31.6% (6/19) against cefotaxime, 31.6% (6/19) against gentamicin, 26.3% (5/19) against amikacin, 21.1% (4/19) against colistin, 15.8% (3/19) against ciprofloxacin, 10.5% (2/19) against azithromycin, 10.5% (2/19) against cefoxitin, and 5.3% (1/19) against imipenem (Fig. 4).

**Fig. 5:**
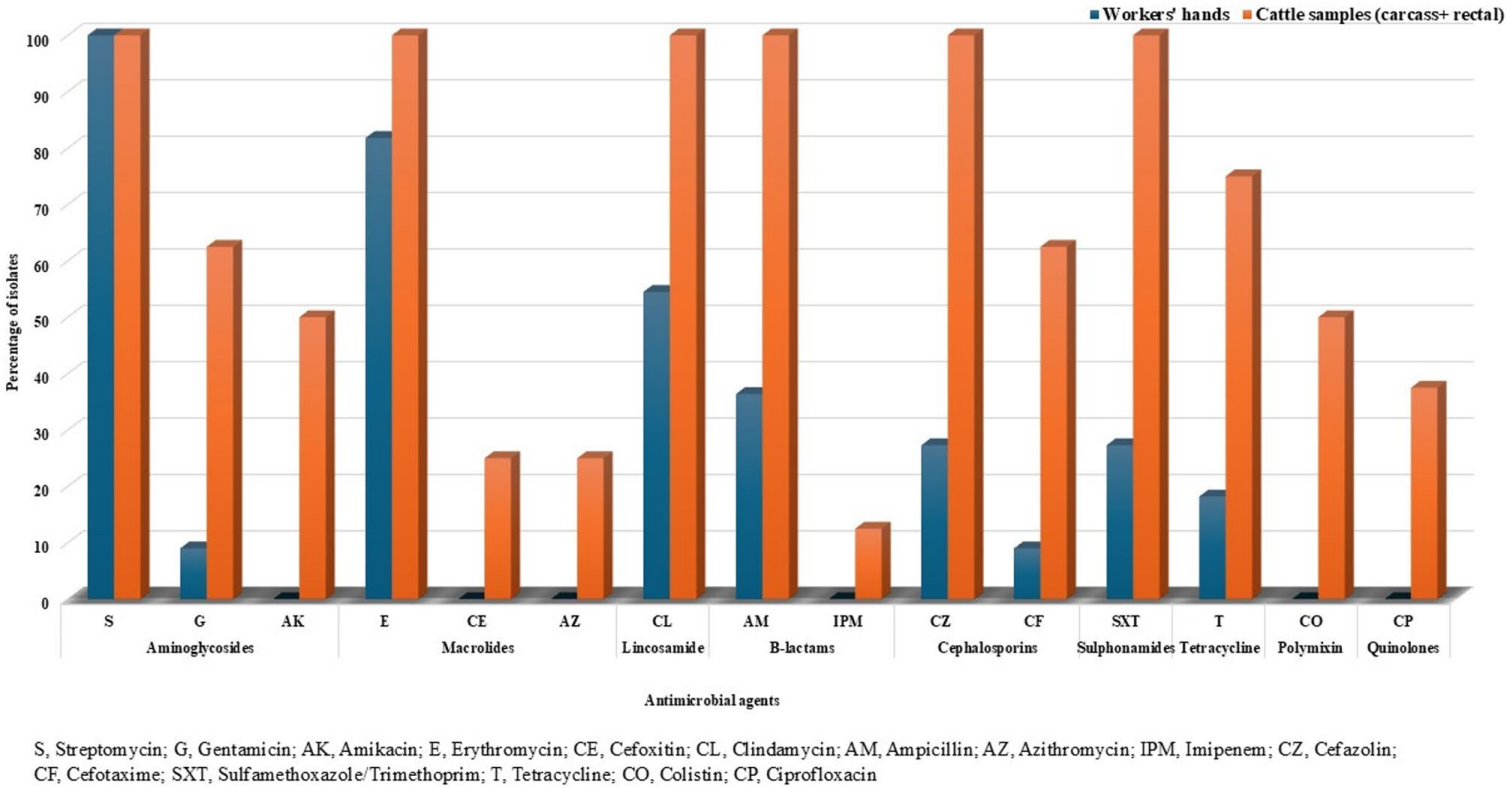
Antimicrobial resistance profiles of *Salmonella* isolates (*n* = 19) recovered from cattle and human

The study detected significant antimicrobial resistance in *Salmonella enterica* serovars from cattle (carcass/rectal swabs) and workers’ hand swabs, especially against streptomycin, erythromycin, clindamycin, ampicillin, cefazolin, and sulfamethoxazole. Additionally, intermediate resistance was detected against tetracycline and gentamicin in isolates from both sources. Notably, cattle-derived *Salmonella* isolates exhibited resistance to amikacin, cefoxitin, azithromycin, imipenem, colistin, and ciprofloxacin (Fig. 5).

### Antibiogram and multiple antibiotic resistance (MAR) index of *Salmonella* isolates

The antimicrobial susceptibility profile of *Salmonella* isolates (*n* = 19) against 16 antimicrobial agents from 9 different antibiotic classes revealed various antimicrobial resistance patterns. Notably, 89.5% (17/19) of the *Salmonella* isolates were resistant to at least two antimicrobial agents. The *Salmonella* isolates were classified based on their antibiotic resistance phenotypes into the following categories: pandrug-resistant (PDR) 5.3% (1/19), extensively drug-resistant (XDR) 5.3% (1/19), multidrug-resistant (MDR) 63.1% (12/19), and low drug-resistant 26.3% (5/19). Among the *Salmonella* isolates, a PDR isolate of *S*. Enteritidis (isolated from cattle carcass) demonstrated resistance to all tested antimicrobial classes, yielding a multiple antibiotic resistance (MAR) index of 1. In contrast, an XDR isolate of *S*. Typhimurium isolated from a rectal swab was classified as extensively drug-resistant, with a MAR index of 0.937 (Table 3).

**Table 3:** Antimicrobial resistance patterns, multiple antibiotic resistance (MAR) indices, and categorization of *Salmonella* isolates (*n* = 19) according to their resistance profiles against 16 tested antimicrobial agents

Isolates	Source of Samples	Antimicrobial resistance profile	Number of antimicrobial classes	MAR index	Classification of antimicrobial resistance phenotypes	
					Type of resistance	No. and Percent of resistance
***S.*** **Enteritidis**	cattle carcass swabs	S, E, CL, AM, CZ, SXT, N, T, G, CF, AK, CO, CP, CE, AZ, IPM	9	1	Pan-drug-resistant	1 (5.3%)
***S***. **Typhimurium**	Rectal swabs	S, E, CL, AM, CZ, SXT, N, T, G, CF, AK, CO, CP, CE, AZ	9	0.937	Extensively drug-resistant	1 (5.3%)
***S***. **Enteritidis**	Rectal swabs	S, E, CL, AM, CZ, SXT, N, T, G, CF, AK, CO, CP	9	0.812	Multidrug -resistance	12(63.1%)
***S***. **Infantis**	cattle carcass swabs	S, E, CL, AM, CZ, SXT, N, T, G, CF, AK, CO	8	0.75		
***S***. **Enteritidis**	Rectal swabs	S, E, CL, AM, CZ, SXT, N, T, G, CF, AK	7	0.687		
***S***. **Typhimurium**	Workers’ hands	S, E, CL, AM, CZ, SXT, N, T, G, CF	7	0.625		
***S***. **Kentucky**	Rectal swabs	S, E, CL, AM, CZ, SXT, N, T	7	0.5		
***S***. **Typhimurium**	Workers’ hands	S, E, CL, AM, CZ, SXT, N, T	7	0.5		
***S***. **Infantis**	Rectal swabs	S, E, CL, AM, CZ, SXT, N	6	0.437		
***S***. **Tsevie**	Rectal swabs	S, E, CL, AM, CZ, SXT	6	0.375		
***S***. **Enteritidis**	Workers’ hands	S, E, CL, AM, CZ, SXT	6	0.375		
***S***. **Paratyphi A**	Workers’ hands	S, E, CL, AM	4	0.25		
***S***. **Enteritidis**	Workers’ hands	S, E, CL	3	0.188		
***S***. **Typhimurium**	Workers’ hands	S, E, CL	3	0.188		
***S***. **Infantis**	Workers’ hands	S, E	2	0.125	Low drug-resistant	5 (26.3%)
***S***. **Kentucky**	Workers’ hands	S, E	2	0.125		
***S***. **Virchow**	Workers’ hands	S, E	2	0.125		
***S***. **Tsevie**	Workers’ hands	S	1	0.063		
***S***. **Haifa**	Workers’ hands	S	1	0.063		

### Detection of virulence and antibiotic resistance genes among *Salmonella* isolates

**Fig. 6:**
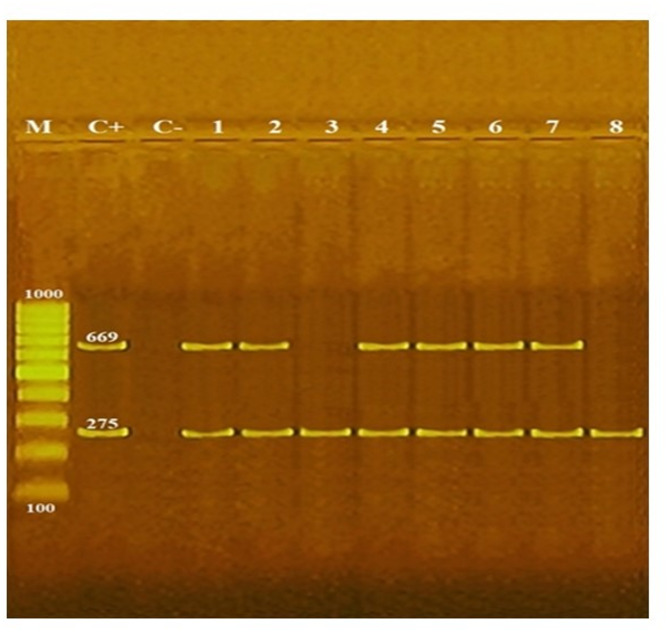
Representative agarose gel electrophoresis of multiplex PCR for virulence genes detection, including *invA* and *spvC* in *Salmonella* serovars isolated from human and cattle samples; Lane M: 100 bp DNA marker; lane C+: positive control for *invA* (275 bp), *spvC* (669 bp); Lane C-: negative control. Lane 1: *S*. Enteritidis (*invA* and *spvC* positive); Lane 2: *S*. infantis (*invA* and *spvC* positive); Lane 3: *S*. Haifa (*invA* positive); Lane 4: *S*. Kentucky (*invA* and *spvC* positive); Lane 5: *S*. Paratyphi A (*invA* and *spvC* positive); Lane 6: *S*. Tsevie (*invA* and *spvC* positive); Lane 7: *S*. Typhimurium (*invA* and *spvC* positive); Lane 8: *S*. Virchow (*invA* positive)

**Fig. 7:**
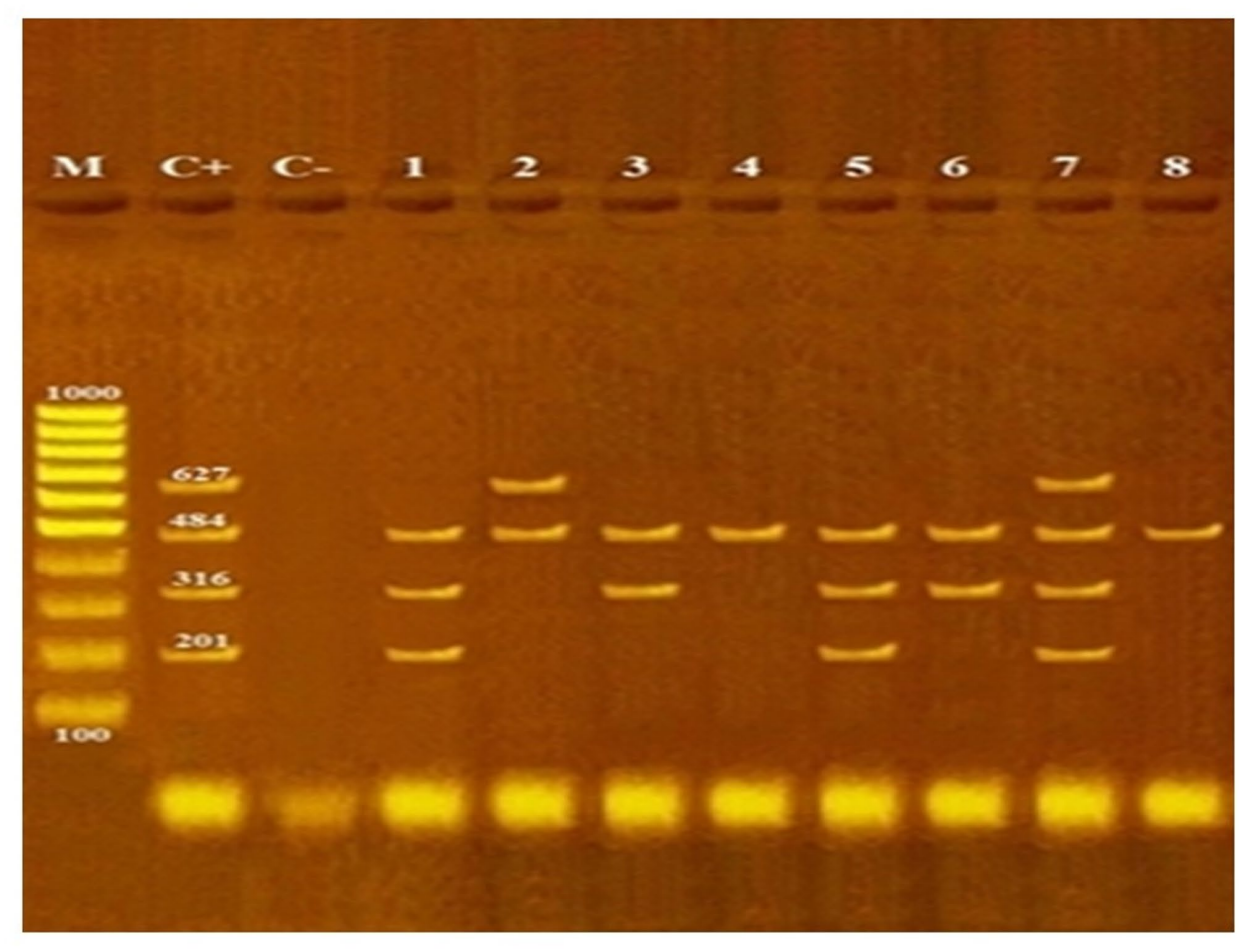
Representative agarose gel electrophoresis for multiplex PCR detection of *tetA*, *sul1*, *aadA1* and *aac(3)-IV* in *Salmonella* serovars isolated from human and cattle samples; Lane M: 100 bp DNA marker; lane C+: positive control for *tetA* (201 bp), *sul1* (316 bp), *aadA1* (484 bp) and *aac(3)-IV* (627 bp); Lane C-: negative control. Lane 1: *S*. Enteritidis (*tetA*, *sul1* and *aadA1* positive); Lane 2: *S*. Infantis (*aadA1* and *aac(3)-IV* positive); Lane 3: *S*. Tsevie (*sul1* and *aadA1* positive positive); Lane 4: *S*. Haifa (*aadA1* positive); Lane 5: *S*. Enteritidis (*tetA*, *sul1* and *aadA1* positive); Lane 6: *S*. Kentucky (*sul1* and *aadA1* positive positive); Lane 7: *S*. Typhimurium (*tetA*, *sul1*, *aadA1* and *aac(3)-IV* positive); Lane 8: *S*. Virchow (*aadA1* positive)

**Fig. 8:**
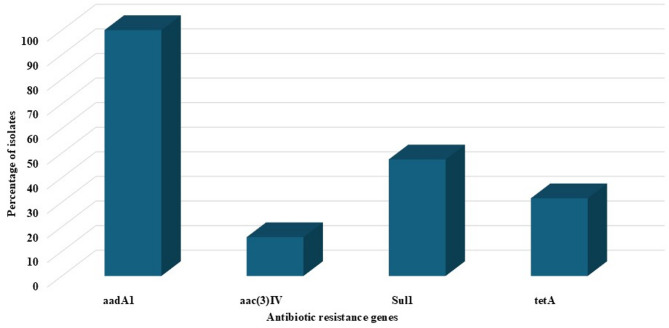
The prevalence of antibiotic resistance genes of *Salmonella* isolates (*n* = 19) across all samples

All tested *Salmonella* isolates 100% (19/19) carried the *invA* virulence gene, while the *spvC* gene was present in 89.5% (17/19) of the isolates (Fig. 6). All *Salmonella* isolates were tested for the presence of antimicrobial resistance genes, including *aadA1*,* aac(3)-IV*,* tetA*, and *sul1* (Fig. 7). The *aadA1* gene was the most prevalent, detected in 100% (19/19) of the isolates, followed by *sul1* and *tetA* at rates of 47.4% (9/19) and 31.6% (6/19), respectively. The *aac(3)-IV* gene was identified as the least prevalent, present in 15.8% (3/19) of the isolates (Fig. 8). The result revealed a clear correlation between antimicrobial resistance genotypes and phenotypes in the *Salmonella* isolates. All 19 isolates carried the *aadA1* gene and demonstrated streptomycin resistance. Additionally, gentamicin resistance was observed in the 3 isolates harboring the *aac(3)-IV* gene, while tetracycline resistance was associated with the presence of the *tetA* gene in 6 isolates. Similarly, sulfonamide resistance was consistently detected in the 9 isolates positive for the *sul1* gene. These findings demonstrate a strong genotype-phenotype relationship for antimicrobial resistance in the studied *Salmonella* population (Table 4).

**Table 4:** The relationship between the phenotypic and genotypic profiles of resistant *Salmonella enterica* isolates (*n* = 19) isolated from cattle and human

Salmonella isolates	sample	Phenotypic antimicrobial resistance	Genotypic antimicrobial resistance	Virulence genes
*S*. Enteritidis	cattle carcass swabs	S, E, CL, AM, CZ, SXT, N, T, G, CF, AK, CO, CP, CE, AZ, IPM	*aadA1*,* tetA*,* sul1*	*invA*,* spvC*
*S*. Typhimurium	Rectal swabs	S, E, CL, AM, CZ, SXT, N, T, G, CF, AK, CO, CP, CE, AZ	*aadA1*,* tetA*,* sul1*,* aac(3)-IV*	*invA*,* spvC*
*S*. Enteritidis	Rectal swabs	S, E, CL, AM, CZ, SXT, N, T, G, CF, AK, CO, CP	*aadA1*,* tetA*,* sul1*	*invA*,* spvC*
*S*. Infantis	cattle carcass swabs	S, E, CL, AM, CZ, SXT, N, T, G, CF, AK, CO	*aadA1*,* aac(3)-IV*	*invA*,* spvC*
*S*. Enteritidis	Rectal swabs	S, E, CL, AM, CZ, SXT, N, T, G, CF, AK	*aadA1*,* tetA*,* sul1*	*invA*,* spvC*
*S*. Typhimurium	Workers’ hands	S, E, CL, AM, CZ, SXT, N, T, G, CF	*aadA1*,* tetA*,* sul1*,* aac(3)-IV*	*invA*,* spvC*
*S*. Kentucky	Rectal swabs	S, E, CL, AM, CZ, SXT, N, T	*aadA1*,* sul1*	*invA*,* spvC*
*S*. Typhimurium	Workers’ hands	S, E, CL, AM, CZ, SXT, N, T	*aadA1*,* tetA*,* sul1*	*invA*,* spvC*
*S*. Infantis	Rectal swabs	S, E, CL, AM, CZ, SXT, N	*aadA1*	*invA*,* spvC*
*S*. Tsevie	Rectal swabs	S, E, CL, AM, CZ, SXT	*aadA1*,* sul1*	*invA*,* spvC*
*S*. Enteritidis	Workers’ hands	S, E, CL, AM, CZ, SXT	*aadA1*,* sul1*	*invA*,* spvC*
*S*. Paratyphi A	Workers’ hands	S, E, CL, AM	*aadA1*	*invA*,* spvC*
*S*. Enteritidis	Workers’ hands	S, E, CL	*aadA1*	*invA*,* spvC*
*S*. Typhimurium	Workers’ hands	S, E, CL	*aadA1*	*invA*,* spvC*
*S*. Infantis	Workers’ hands	S, E	*aadA1*	*invA*,* spvC*
*S*. Kentucky	Workers’ hands	S, E	*aadA1*	*invA*,* spvC*
*S*. Virchow	Workers’ hands	S, E	*aadA1*	*invA*
*S*. Tsevie	Workers’ hands	S	*aadA1*	*invA*,* spvC*
*S*. Haifa	Workers’ hands	S	*aadA1*	*invA*

## Discussion

Non-typhoidal *Salmonella* (NTS) is a prevalent foodborne pathogen frequently carried by asymptomatic cattle, posing contamination risks during slaughter and processing [30–32]. In this study, samples from an Egyptian abattoir with poor hygiene practices revealed a 12.7% (19/150) *Salmonella* prevalence, with the highest contamination on workers’ hands (22%, 11/50), followed by rectal swabs (12%, 6/50) and cattle carcasses (4%, 2/50). In this abattoir, the lack of proper sanitation, including processing at room temperature and mixing live animals with dressed meat, likely contributed to bacterial spread. These findings highlight the critical need for improved hygiene standards in abattoirs to reduce NTS transmission through the food chain.

Workers’ hands in abattoirs serve as a major transmission route for *Salmonella*, posing significant food safety and public health risks. Our study revealed a 22% (11/50) contamination rate on workers’ hands, indicating a high carrier rate and potential spread to carcasses. This prevalence exceeds earlier reports from Egypt (8%, 4/50) [33] and Nigeria (1.6%, 1/63) [34], highlighting elevated hygiene concerns. These findings underscore the urgent need for stricter hand hygiene protocols in abattoirs to mitigate *Salmonella* transmission. The study detected *Salmonella* in 12% (6/50) of rectal swabs, indicating potential pre-slaughter herd infection, higher than previous reports of 5.6% (11/195) [35], 7.1% (5/70) [36], and 8.5% (5/59) [37]. Our study found a relatively low *Salmonella* prevalence in cattle carcasses (4%, 2/50), consistent with Egypt’s reported 3.33% [38] but significantly lower than rates in Ethiopia (8%, 25/312) [39], Egypt (20%, 5/25) [40], South Africa (30%, 30/100) [41], and Ghana (75%, 45/60) [42]. Conversely, our rates exceeded those from the Republic of Ireland (0.25%, 1/400) [43], Germany (0.7%, 29/4,170) [44], South Korea (2.04%, 1/49) [45], and Ethiopia (2.4%, 6/250) [46]. These differences reflect geographic, hygienic, and operational factors, such as transport stress and slaughter methods that collectively affect contamination risks [47].

Our study identified seven NTS serovars, including *S*. Enteritidis, *S*. Typhimurium, *S*. Infantis, *S*. Kentucky, *S*. Tsevie, *S*. Haifa, and *S*. Virchow. *S*. Enteritidis showed the highest prevalence across samples (carcasses: 5.3%, 1/19; rectal contents: 10.5%, 2/19; workers’ hands: 10.5%, 2/19), exceeding rates reported by Adel et al. (2021) [48] but lower than Abd-Elghany et al. (2022) [49], Mansour et al. (2023) [33], and Tawfik et al. (2022) [50]. Notably, *S*. Typhimurium accounted for 15.8% (3/19) of workers’ hands, exceeding previous reports [33]. Our study also revealed three previously unreported serotypes (*S*. Infantis, *S*. Kentucky, *S*. Tsevie) on workers’ hands in Egyptian abattoirs [33]. These distinct serotype patterns underscore the importance of facility-specific *Salmonella* monitoring in food production environments. In the current study, *S*. Virchow and *S*. Haifa were the least prevalent serotypes (5.3% (1/19) each) on workers’ hands, aligning with Mansour et al.’s findings [33]. A recent case report described severe *S*. Paratyphi A infection leading to multi-organ failure [51]. *S*. Paratyphi A was also detected on 5.3% (1/19) of workers’ hands, indicating poor hygiene and potential infection risks, especially with hand injuries. These findings highlight the need for improved hygiene practices in abattoirs to prevent *Salmonella* transmission.

### Antibiogram of *Salmonella enterica* serovars

In veterinary practice, antibiotics are widely used not only for treating infections but also as growth enhancers and to optimize feed efficiency and weight gain in animals. In both animal and human populations, the excessive and often inappropriate use of antibiotics leads to increase the antibiotic resistance [52]. *Salmonella* is one of the most frequently reported zoonotic pathogens, and the emergence of antimicrobial-resistant (AMR) strains poses a significant public health concern [53].

The high levels of antimicrobial resistance observed among *Salmonella enterica* serovars isolated from cattle (cattle carcass swab and rectal swab) and human (workers hands swab) in this study, particularly toward streptomycin, erythromycin, clindamycin, ampicillin, cefazolin, sulfamethoxazole, indicating that these antibiotics are likely to be extensively used in veterinary medicine. This widespread resistance pattern suggests that these drugs may be frequently administered to animals, either for therapeutic purposes, as growth promoters, or for prophylactic measures. Such practices create selective pressure, enabling resistant bacterial strains to survive and proliferate. However, findings from other studies highlight diverse resistance patterns. For example, a previous study identified ampicillin, streptomycin, oxacillin, and tetracycline as the most prevalent resistance profiles in *Salmonella* isolates obtained from retail meat and beef carcasses [54]. Another study focusing on *Salmonella* serovars isolated from slaughtered cows reported high resistance rates: rifampicin and clindamycin at 100% (43/43), ampicillin at 81% (35/43), cefepime at 42% (31/43), and cephalexin at 37% (16/43) [55]. In contrast, a separate study analyzing *Salmonella* isolates from buffalo meat found resistance rates of 100% (53/53) for erythromycin, 98.1% (52/53) for streptomycin, 94.3% (50/53) for clindamycin, 77.4% (41/53) for cefepime, and 66% (35/53) for nalidixic acid [49]. These variations in resistance patterns likely reflect differences in antibiotic usage practices, geographical locations, and sources of *Salmonella* isolates. The detection of high antibiotic resistance in *Salmonella* serovars isolated from both cattle and humans strongly suggests the occurrence of cross-transmission between these hosts during cattle evisceration. This finding highlights the interconnected nature of human and animal health, demonstrating how the transmission of *Salmonella* can be facilitated through direct contact. Such cross-transmission not only complicates efforts to control infections but also increases the risk of spreading resistant strains, further exacerbating public health challenges [56].

Surprisingly, in the present study, 50% (4/8) of *S.* Enteritidis isolates from cattle exhibited resistance to colistin. Among these resistant isolates, *S.* Enteritidis emerged as the predominant serovar associated with colistin resistance. Similarly, the European Food Safety Authority and European Centre for Disease Prevention and Control reported that Colistin resistance was notably high in *S*. Enteritidis isolated from food-producing animals with an average incidence of 54.25% [57]. Colistin, a member of the polymyxin class of antibiotics, is considered a last choice antibiotic due to its effectiveness against multidrug-resistant (MDR) Gram-negative pathogens. However, its use in veterinary medicine has raised significant concerns. The administration of colistin in animals can lead to the selection and acquisition of colistin resistance in zoonotic bacterial strains, such as non-typhoidal serovars of *Salmonella enterica*. As a result, resistance mechanisms can spread from animals to humans. This underscores the critical need for stringent regulation and judicious use of colistin in veterinary practices to preserve its efficacy for treating severe infections in humans [58].

In this study, *Salmonella enterica* isolates from cattle demonstrated high resistance rates, with 62.5% (5/8) showing resistance to both gentamicin and cefotaxime. Comparatively, a study of *Salmonella* isolates from slaughtered cattle in Nigeria reported even higher resistance to gentamicin at 84.6% (11/13), while resistance to cefotaxime was lower at 20% (1/13) [59]. Additionally, in this study, *Salmonella enterica* isolates from cattle exhibited resistance rates of 37.5% (3/8) to ciprofloxacin and 25% (2/8) to azithromycin. In contrast, the Nigerian study found resistance rates of 30.8% (4/13) to ciprofloxacin and 53.8% (7/13) to azithromycin among *Salmonella* isolates from slaughtered cattle [59].

Amikacin, an aminoglycoside antibiotic, is extensively used to treat severe Gram-negative bacterial infections in veterinary medicine, including those caused by *Salmonella* [60]. However, due to its widespread use, 50% (4/8) of *Salmonella enterica* serovars isolates from cattle in this study exhibited resistance to amikacin. In comparison, resistance rates to amikacin were significantly higher in *Salmonella* isolates from abattoirs in Egypt at 76.9% (10/13) [33], while much lower in isolates from Ethiopia at 4.76% (1/21) [61]. Regarding tetracycline, *Salmonella* isolates in this study showed resistance rates of 18.2% (2/11) in human isolates and 100% (2/2) in cattle carcass isolates. Conversely, in Ethiopia, tetracycline resistance rates were 100% (2/2) in human isolates and 56% (14/25) in carcass isolates from abattoirs [39]. In this study, imipenem, a carbapenem antibiotic, exhibited a resistance rate of 50% (1/2) in *S*. Enteritidis isolates obtained from cattle carcasses. This finding is particularly concerning, as carbapenems like imipenem are often considered last-resort antibiotics for treating severe infections caused by multidrug-resistant bacteria. In contrast, earlier studies conducted in Nigeria reported that all *Salmonella* isolates were susceptible to imipenem, highlighting a significant shift in resistance patterns across regions [59, 62].

### Distribution of virulence genes among *Salmonella* serovars

This study detected two key virulence genes in *Salmonella* isolates: *invA* was present in all samples (100%, 19/19), while *spvC* appeared in 89.5% (17/19). These results contrast with prior studies, for instance Naidoo et al. [63] found *invA* in all South African beef samples but no *spvC*, while Beyene et al. [64] reported *invA* in all isolates from Ethiopian milkers but *spvC* only in animal feces. Kang et al. [65] found no *Salmonella* in Ethiopian cattle meat, whereas another Ethiopian study reported *invA* in just 16.7% of beef carcasses and workers’ hands [66]. The *invA* gene facilitates intestinal cell invasion, while *spvC* promotes intracellular survival, systemic spread, and immune evasion [11]. Given these virulence mechanisms, the high prevalence of both genes in Egyptian cattle, carcasses, and workers highlights significant public health risks, demanding rigorous surveillance to curb transmission through livestock and food chains.

### Distribution of aminoglycosides, tetracyclines and sulfonamides resistance genes among *Salmonella* serovars

*Salmonella* develops antibiotic resistance through chromosomal or plasmid-encoded genetic determinants that drive intrinsic mechanisms like beta-lactamase production, antimicrobial enzymatic modification, membrane permeability changes, efflux pumps, or altered target receptors [67]. Acquired resistance emerges via chromosomal mutations or mobile genetic elements (plasmids, transposons, genomic islands), collectively fostering AMR development [67]. Notably, resistance commonly spans broad-spectrum antibiotics including aminoglycosides, tetracyclines, and sulfonamides [68]. Such resistance traits are frequently plasmid-mediated, enhancing their spread across bacterial populations [69]. These multifactorial mechanisms underscore the complexity of combating resistant *Salmonella* strains.

Resistance of *Salmonella* to aminoglycosides is driven by the enzymatic inactivation of the antibiotics [70]. The genes encoding aminoglycoside-modifying enzymes are frequently found on mobile genetic elements, including plasmids and transposons. This genetic mobility facilitates their transfer between bacterial species, enabling the rapid spread of aminoglycoside resistance [71]. This resistance is attributed to the presence of *aadA1* and *aac(3)-VIa* genes which encode enzymes responsible for conferring resistance to streptomycin and gentamicin, respectively [72, 73]. In this study, the *aadA1* gene was detected in all *Salmonella* isolates (100%), whereas the *aac(3)-VIa* gene was present in 15.8% of the isolates. A former study in Egypt revealed that *aadA1* gene was detected in 100% of MDR *Salmonella* isolates from poultry and food products [74, 75].

Tetracyclines are a class of broad-spectrum antibiotics that are effective against a diverse range of bacterial species. However, their widespread use has contributed to the emergence of resistance, particularly in *Salmonella* species. This resistance is primarily attributed to the presence of *tet* genes (genes encoding tetracycline efflux pump) within these bacteria. In this study, the *tetA* gene was found in 31.6% (6/19) of *S*. Enterica serotypes, including Enteritidis and Typhimurium, isolated from both cattle and human sources, aligning with findings from previous researches [16]. Additionally, the *tetA* gene was detected in 4.5% (1/22) of *Salmonella enterica* isolates from cattle and human samples collected at an abattoir in Nigeria. This particular isolate belonged to a rare serovar known as *S.* Concord [59].

Sulfonamides represent a group of broad-spectrum antimicrobial agents extensively used in both human healthcare and veterinary practices. In Gram-negative bacteria, resistance to sulfonamides is primarily driven by the presence of *sul* genes. Notably, *sul1* and *sul2* have been commonly detected in *Enterobacteriaceae*, with a particular prevalence in genera such as Escherichia and *Salmonella* [76]. In this study, the *sul1* gene was identified in 47.4% (9/19) of the *Salmonella* isolates. This finding aligns with previous studies, which have demonstrated that *Salmonella* species exhibit a significantly high prevalence of resistance to both tetracycline and sulfonamide antibiotics. These studies conducted in various countries, including Egypt, Canada, Korea, Chile, South Africa, Poland, Iran, Vietnam, and Brazil, have reported resistance rates for tet genes ranging from 20.6 to 87.7% and for *sul1* genes ranging from 8 to 90.5%, depending on the source and region [16]. These variations highlight the widespread and diverse nature of antimicrobial resistance in *Salmonella*, underscoring the need for global surveillance and targeted interventions to combat this issue.

### Multidrug-resistant (MDR) pattern

The emergence of multidrug-resistant (MDR) *Salmonella* serovars is reducing treatment efficacy and may increase mortality rates [77]. In our study, we identified 14 drug-resistant isolates: 1 PDR, 1 XDR and 12 MDR of different *Salmonella* serotypes, harboring multiple resistance genes (*aadA1*, *aac(3)-IV*, *tetA*, *sul1*). Similarly, most of the *S*. Enteritidis and *S*. Typhimurium serovars recovered from broilers in Egypt were XDR to several classes and carried *sul1*, *tetA*, and *aadA1* genes [78]. Recent reports confirm the alarming spread of MDR *Salmonella* across various sources in Egypt [48, 79–82], underscoring the urgent need for comprehensive surveillance in human, animal, food, and environmental sectors to track resistance patterns and guide treatment strategies. Our study found that 63.15% (12/19) of *Salmonella* isolates exhibited a Multiple Antibiotic Resistance (MAR) index ≥ 0.2, signaling substantial antibiotic overuse in both animal and human populations [83]. This high resistance prevalence demonstrates these *Salmonella* isolates repeated exposure to diverse antimicrobials, facilitating multi-drug resistance development. These findings particularly highlight the urgent need for stricter antibiotic use regulations in livestock production.

## Conclusion

Our study identified alarming resistance rates in Egyptian *Salmonella* isolates from cattle and humans: 5.3% PDR, 5.3% XDR, and 63.1% MDR, with 100% carrying *invA* and 89.5% *spvC* virulence genes. The predominance of resistant *S*. Enteritidis and *S*. Typhimurium isolates in the food chain poses serious treatment challenges. These findings demand immediate action, including strict antibiotic regulation in livestock and enhanced surveillance systems. Further research is crucial to understand resistance transmission and develop effective control measures against these virulent, drug-resistant isolates.

## Electronic supplementary material

Below is the link to the electronic supplementary material.

Supplementary Material 1

Supplementary Material 2

## Data Availability

No datasets were generated or analysed during the current study.

## Abbreviations

AMR: Antimicrobial resistance
DNA: Deoxyribonucleic acid
MAR: Multiple Antibiotic Resistance
PCR: Polymerase Chain Reaction
XLD: Xylose-lysine-deoxycholate
invA: Invasion A
spvC: Salmonella plasmid virulence C
WHO: World Health Organization
MDR: Multi-drug resistant
PDR: Pan-drug resistant
XDR: Extensively drug resistant
