# Supplementary figures and images for: Pan-drug, colistin, streptomycin, erythromycin, clindamycin resistant Salmonella enterica serovars isolated from slaughtered cattle and human in mansoura, Egypt
Source: Ann Clin Microbiol Antimicrob. 2025 Jul 3;24:40. doi: 10.1186/s12941-025-00809-4 (PMC12224357; doi:10.1186/s12941-025-00809-4)

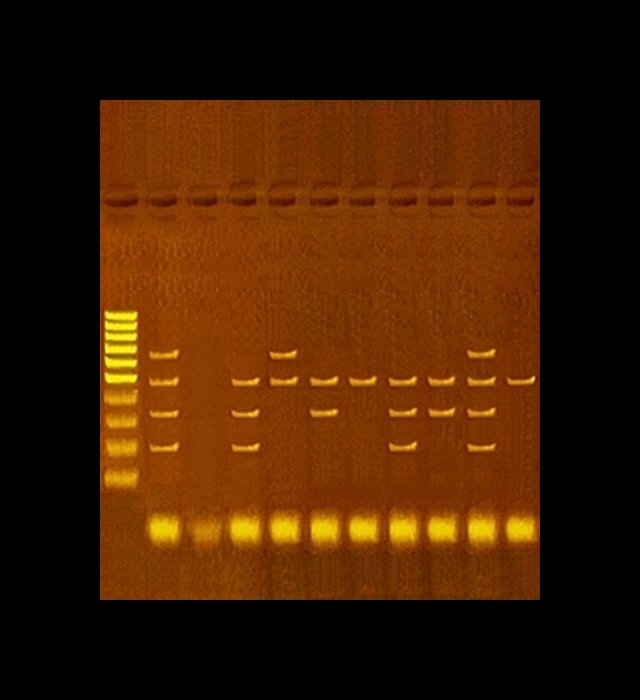

Supplement: Supplementary file 1 — Supplementary Material 1 [file 12941_2025_809_MOESM1_ESM.jpg]

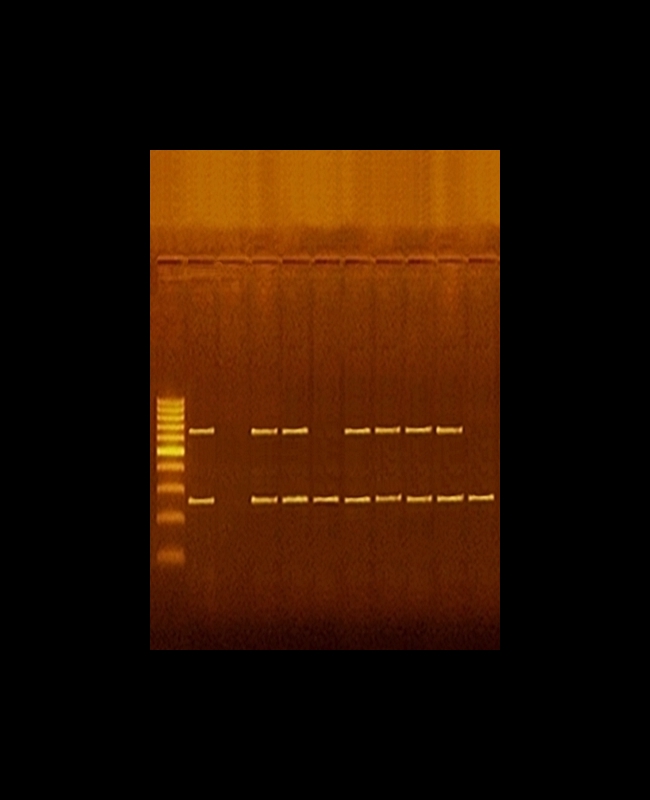

Supplement: Supplementary file 2 — Supplementary Material 2 [file 12941_2025_809_MOESM2_ESM.jpg]
